# On the saliva proteome of the Eastern European house mouse (*Mus musculus musculus*) focusing on sexual signalling and immunity

**DOI:** 10.1038/srep32481

**Published:** 2016-08-31

**Authors:** Pavel Stopka, Barbora Kuntová, Petr Klempt, Leona Havrdová, Martina Černá, Romana Stopková

**Affiliations:** 1 BIOCEV group, Department of Zoology, Faculty of Science, Charles University in Prague, Viničná 7, CZ 12844, Czech Republic

## Abstract

Chemical communication is mediated by sex-biased signals abundantly present in the urine, saliva and tears. Because most studies concentrated on the urinary signals, we aimed to determine the saliva proteome in wild *Mus musculus musculus*, to extend the knowledge on potential roles of saliva in chemical communication. We performed the gel-free quantitative LC-MS/MS analyses of saliva and identified 633 proteins with 134 (21%) of them being sexually dimorphic. They include proteins that protect and transport volatile organic compounds in their beta barrel including LCN lipocalins, major urinary proteins (MUPs), and odorant binding proteins (OBPs). To our surprise, the saliva proteome contains one MUP that is female biased (MUP8) and the two protein pheromones MUP20 (or ‘Darcin’) and ESP1 in individuals of both sex. Thus, contrary to previous assumptions, our findings reveal that these proteins cannot function as male-unique signals. Our study also demonstrates that many olfactory proteins (e.g. LCNs, and OBPs) are not expressed by submandibular glands but are produced elsewhere–in nasal and lacrimal tissues, and potentially also in other oro-facial glands. We have also detected abundant proteins that are involved in wound healing, immune and non-immune responses to pathogens, thus corroborating that saliva has important protective roles.

The sequence of the mouse genome provided a tool to study blueprints for all RNAs and proteins in mice^1^. Progenitors of modern laboratory mice were hybrids among *Mus musculus domesticus*, *Mus musculus musculus* and other subspecies. Though, laboratory mice have been widely and successfully used as experimental organisms in studies of biomarkers of physiological states^2^ and of human pathological conditions, they may be less suitable to study chemical communication, a process which is driven by sexual selection. This is due to the differential contribution of blocks of genes from the two house-mouse subspecies *M. m. domesticus* and *M. m. musculus* to current laboratory strains^1^ that may mask typical intra- and inter-specific differences. One of our aims, therefore, was to define the saliva proteome in *M. m. musculus* to provide the array of proteins and their quantity characteristic for saliva in a wild living mouse species. This study also represents a baseline for future comparative studies focusing on chemical communication and immunity.

In mice, the most published studies in chemical communication focused on the major urinary proteins (MUPs)^3^,^4^,^5^,^6^,^7^, which are expressed by the liver and transport volatile organic compounds (VOCs) in their beta barrel structure to the urine^6^,^7^,^8^,^9^,^10^,^11^. VOCs are slowly released from different urinary MUPs, and have been proposed to function in a variety of social signals, including identity, territorial marking, mate choice etc.^3^,^12^,^13^,^14^. Thus, lipocalins and their specific ligands together form a signal^15^. Differential ligand binding may have a potential influence on sub-species recognition between *M. m. musculus* and *M. m. domesticus*^16^,^17^,^18^. These two sub-species have been previously shown to vary in the abundance of male VOCs^19^ and in MUP expression between the two subspecies and individuals of the opposite sex^9^. Moreover, scent signals have been shown to be an integral part of subspecies recognition and could play important roles in preventing interspecific mating between the two house mouse subspecies^18^.

Increasing number of papers, however, show that MUP expression is linked to reproduction and sociality, and not just to competitive ability^3^,^4^,^20^,^21^,^22^,^23^. The expression of urinary MUPs is socially regulated in that males excrete higher quantities of MUPs in contacts with females in the laboratory mouse^23^ as well as in wild *M. m. musculus*^21^, and in *M. m. domesticus*^3^. Furthermore, MUPs have a predictive value for the onset of aggressive behaviour and dispersal tendency in male wild house mice^24^ and it is evident that scent marking signals have strong effects on the reproductive success of the signaller^20^. Furthermore, amongst MUPs, MUP20 (or ‘Darcin’) has been reported to predict the outcome of male-male territorial competition^3^.

MUPs are products of a gene cluster that contains 21 coding genes (and a similar number of pseudogenes), and can be divided into two groups, the group-A (ancestral), containing *Mup3, Mup4, Mup5, Mup6, Mup20* and *Mup21*, and the group-B, consisting of 15 other *Mup*s (i.e. *Mup1, Mup2, Mup7-Mup19*) sharing almost 99% sequence identity^25^,^26^, reviewed in refs 22,27. Different MUPs were originally supposed to present an individual ‘barcode’ signal^13^ by which different individuals recognize each other. However, a recent study with a sufficient sample size shows that the urinary profiles of wild male house mice *M. m. musculus* are not individually unique but are dynamic over time with significant changes after puberty and during adulthood^28^. Moreover, the variation in pheromone affinities of the urinary MUP isoforms provides low support for the proposal that heterogeneity in MUPs plays a role in regulating profiles of available pheromones^10^. However, MUPs have also been reported in several tissues other than the liver including salivary glands, olfactory/vomeronasal epithelia, and nasal-associated lymphoid tissues^2^,^8^,^29^,^30^,^31^, but their functions are not yet fully understood.

Another interesting group of lipocalins involved in chemical communication are products of the odorant binding protein genes (*Obp*). The X-linked *Obp* genes were thought to involve just two nasal members–*Obp1a*, and *Obp1b*^32^. However, *Obp* genes have undergone a series of duplications in mice, and they occur in a cluster of seven genes (i.e. including *Prb*, Probasin) and two pseudogenes on the X chromosome^27^,^31^,^33^. All OBPs including Probasin have a specific disulfide bond (Cys38–Cys42), which represents a strong OBP-diagnostic motif CXXXC - Cys-Xaa-Xaa-Xaa-Cys^27^,^33^. To date, *Obp*s/OBPs were detected in various mammalian taxa, e.g., house mice^31^, bank voles^34^, porcupines^35^, elephants^36^, cows^37^, and boar^38^,^39^. Interestingly, pigs have OBPs and SAL. SAL is the major salivary protein in pigs with affinity to steroids and to 2-isobutyl-3-methoxypyrazine, it is phylogenetically close to MUPs and is expressed by the male submaxillary glands^40^. Furthermore, aphrodisin is an OBP^34^ described as the major pheromone transporter in vaginal flushes of hamsters (*Cricetus cricetus*)^41^.

In our latest study^31^, we described particular mRNA expression sites for the newly described odorant binding proteins in wild mice (*M. m. musculus, M. m. domesticus*). They are highly expressed with other lipocalins (LCNs, MUPs) in the mouse lacrimal, nasal, and vomeronasal tissues with the normalized expression levels being as high or higher as those described for the urinary group-B *Mup* genes in the liver. Lacrimal glands expressed the mRNA coding OBP5, OBP6 and OBP7 whilst the mRNAs coding OBP1/OBP2, OBP5, and OBP7 were highly abundant in the olfactory epithelia (OE), vomeronasal organ (VNO), and nasal-associated lymphoid tissue (NALT) in the both house mouse subspecies. No *Obp* mRNA was detected in submandibular glands but at least one OBP protein (i.e. OBP5) was detected in the saliva. We have also provided evidence that *Obp* transcripts are co-expressed in combination with other lipocalin transcripts (e.g. nasal and lacrimal *Obp5* and *Obp7* with *Mup4* and *Lcn11*), presumably to widen the spectrum of ligands that OBP, MUP, and LCN proteins may sequester and transport^31^. Thus, this study also aims to detect particular OBPs in the mouse saliva to test whether OBPs are involved in the transport of VOCs and various degradation products from the nasal and lacrimal tissues to the oral cavity where digestion starts.

This study was conducted to detect the level of sexual dimorphisms with a particular interest in lipocalins that have potential roles in chemical communication as transporters of salivary VOCs. We used sensitive proteomic techniques to identify proteins and partially also RNAseq on GS Junior to detect a potential expression site for some of the lipocalins of particular interest (i.e. MUP20, OBPs). Because eyes, nose and mouth are primary gates for various pathogens to enter the body and at the same time a route of receiving or transmitting pheromones, it is believed that an organismal detoxification, immunity and chemical communication might have been driven by similar evolutionary forces^27^,^33^,^42^. Thus, we also discuss our results on other protein families significantly enriched in the saliva proteome of the house mouse.

## Materials and Methods

### Ethical Standards

All animal procedures were carried out in strict accordance with the law of the Czech Republic paragraph 17 no. 246/1992 and the local ethics committee of the Faculty of Science, Charles University in Prague specifically approved this study in accordance with the accreditation no. 27335/2013-17214 valid through 2019.

### Animals

To allow for natural variation between samples we selected individuals of similar weight, in reproductive condition, but from different sites: 1M+1F locality Bříza (50.3605111N, 14.2162558E), 1M+3F Velke Bilovice (48.8492886N, 16.8922736E), 3M Bohnice (50.1341539N, 14.4142189E), 1F Dolni Brezany (49.9632106N, 14.4585047E), 1F Bruntal (49.9884447N, 17.4647019E). After the protein sample collection, all experimental individuals were sacrificed by cervical dislocation and tissues were further used for the transcriptome analyses.

### Samples

The saliva samples were collected by gentle flushing with a pipette using 50 μl of the 0.9% saline solution from six female and five male biological replicates, and each sample was analysed twice to produce the mean values from the methodology duplicates. This was done in the ‘in-house’ Mass Spectrometry and Proteomics Service Laboratory, Faculty of Science, Charles University in Prague.

### Protein Digestion

The protein concentration of each lysate was determined using the BCA assay kit (Fisher Scientific). Cysteins in 200 μg of proteins were reduced with the final concentration of 5 mM TCEP (60 °C for 60 min) and blocked with10 mM (10 min Room Temperature). Samples were cleaved with trypsine (i.e. 1/100, trypsine/protein). Peptides were desalted on Michrom C18 column.

### nLC-MS^2^ Analysis

Nano Reversed phase columns were used (EASY-Spray column, 50 cm × 75 μm ID, PepMap C18, 2 μm particles, 100 Å pore size). Mobile phase buffer A was composed of water, 2% acetonitrile and 0.1% formic acid. Mobile phase B contained 80% acetonitrile, and 0.1% formic acid. Samples were loaded onto the trap column (Acclaim PepMap300, C18, 5 μm, 300 Å Wide Pore, 300 μm × 5 mm, 5 Cartridges) for 4 min at 15 μl/min loading buffer was composed of water, 2% acetonitrile and 0.1% trifluoroacetic acid. After 4 minutes ventile was switched and Mobile phase B increased from 2% to 40% B at 60 min, 90% B at 61 min, hold for 8 minutes, and 2% B at 70 min, hold for 15 minutes until the end of run.

Eluting peptide cations were converted to gas-phase ions by electrospray ionization and analysed on a Thermo Orbitrap Fusion (Q-OT-qIT, Thermo). Survey scans of peptide precursors from 400 to 1600 *m*/*z* were performed at 120 K resolution (at 200 *m*/*z*) with a 5 × 10^5^ ion count target. Tandem MS was performed by isolation at 1.5 Th with the quadrupole, HCD fragmentation with normalized collision energy of 30, and rapid scan MS analysis in the ion trap. The MS^2^ ion count target was set to 10^4^ and the max injection time was 35 ms. Only those precursors with charge state 2–6 were sampled for MS^2^. The dynamic exclusion duration was set to 45 s with a 10 ppm tolerance around the selected precursor and its isotopes. Monoisotopic precursor selection was turned on. The instrument was run in top speed mode with 2 s cycles.

### Data analysis

All data were analysed and quantified with the MaxQuant software (version 1.5.3.8)^43^. The false discovery rate (FDR) was set to 1% for both proteins and peptides and we specified a minimum peptide length to seven amino acids. The Andromeda search engine was used for the MS/MS spectra search against the Uniprot *Mus musculus* database (downloaded on June, 2015, containing 44,900 entries. Enzyme specificity was set as C-terminal to Arg and Lys, also allowing cleavage at proline bonds and a maximum of two missed cleavages. Dithiomethylation of cysteine was selected as fixed modification and N-terminal protein acetylation and methionine oxidation as variable modifications.

The “match between runs” feature of MaxQuant was used to transfer identifications to other LC-MS/MS runs based on their masses and retention time (maximum deviation 0.7 min) and this was also used in all quantification experiments. Quantifications were performed with the label-free algorithms described recently^43^.

### Gene ontology analysis

We used the PANTHER (Protein ANalysis THrough Evolutionary Relationships) Classification System to classify proteins according to Biological process, which is the function of the protein in the context of a larger network of proteins that interact to accomplish a process at the level of the tissue^44^. Each analysis involved Bonferroni corrections for multiple testing.

### RNAseq

The submandibular glands were homogenized in RLT buffer (Qiagen) with MagNALyser (Roche) for 30 s at 6000 rpm. We used the RNeasy Mini Kit (Qiagen) for RNA isolation following the manufactures protocol with on-column DNase I treatment. The purity and concentration of eluted RNA was measured with NanoDrop ND1000. The quality of RNA was checked using the agarose gel electrophoresis and pre-selected samples were further analysed with Agilent Bioanalyzer using the RNA Nano 6000 chip to obtain information on the RNA integrity. Treated samples were cleaned using the RNA cleanup procedure (RNeasy Mini Kit) and checks of the quality with AGE and Bioanalyzer were repeated as well as the measurement with NanoDrop. RIN of twelve submandibular gland samples after this procedures ranged from 4.60 to 7.6. We decided to eliminate the worst two samples (i.e. one from female with RIN 4.60, and one from male with RIN 6.00). The remaining ten RNA samples RIN values were following: 4.70, 5.90, 7.00, 6.70, 5.90 for females and 6.40, 6.70, 7.60, 7.30 and 7.10 for males. RNA samples were standardly stored at −80 °C.

cDNA was prepared using the SMARTer PCR cDNA Synthesis Kit (Clontech) and amplified with Advantage 2 PCR Kit (Clontech). Both procedures were handled according to the Trimmer-2 Normalization Kit (Evrogen) protocol. The products of optimalized cDNA amplification were then loaded on AGE. For each sample, only the area of product in range from ~500 bp to ~1300 bp (well visible area full of bands) was excized from the gel and the DNA products were extracted using the Gel/PCR DNA Fragments Extraction Kit (Geneaid). To avoid potential contaminats contaminants we performed AMPure XP cleanup (Beckman-Coulter). Purified products (and the range where they emerge) were checked on AGE. DNA concentration was determined using Quant-it Pico Green dsDNA Assay Kit (Invitrogen) and fluorimeter (Hoefer DQ 300). For each obtained submandibular gland size-selected transcriptomes from 5 males and 5 females we prepared Rapid Libraries (RL) according to Rapid Library Preparation Manual (my454.com). Rapid libraries were checked for the presence of small fragments on BioAnalyzer using the High Sensitivity DNA kit. Equal amount from each of 10 Rapid Libraries (107 molecules per μl dilution) were mixed and then used for emPCR. Further steps were following the provider’s instructions for sequencing with GS Junior+ (Roche; emPCR Amplification Method Manual Lib-L and Sequencing Method Manual, my454.com). In order to reach better sequencing depth we combined two sequencing runs (140 000 HQ reads and 120 000 HQ reads). Both.sff datasets were merged using the sff file tool (part of GS Junior+ Roche system software). Merged reads were then multiplexed, trimmed (i.e. using trimming database that contains primers used for the libraries preparation), filtered and aligned into contigs against *Mus musculus* cDNA database (“the super-set of all known, novel and pseudo gene predictions”; ensembl.org) and using GS Reference Mapper (Roche). Highly detected transcripts are graphically represented in Supplementary Figure 1.

### Phylogenetic analysis by Maximum Likelihood method

The evolutionary history was inferred by using the Maximum Likelihood method based on the JTT matrix-based model^45^. The tree with the highest log likelihood (−1578.6730) is shown. The percentage of trees in which the associated taxa clustered together is shown next to the branches. Initial tree(s) for the heuristic search were obtained automatically by applying Neighbor-Join and BioNJ algorithms to a matrix of pairwise distances estimated using the Maximum Composite Likelihood (MCL) approach, and then selecting the topology with superior log likelihood value. The tree is drawn to scale, with branch lengths measured in the number of substitutions per site. The analysis involved 24 nucleotide sequences from the mouse genome. Codon positions included were 1st+2nd+3rd+Noncoding. All positions with less than 95% site coverage were eliminated. That is, fewer than 5% alignment gaps, missing data, and ambiguous bases were allowed at any position. Evolutionary analyses were conducted in MEGA5^46^. We followed the standard MGI/NCBI nomenclature for all proteins with the exception of OBPs where our recently^31^ submitted *M. m. musculus* names/synonyms were used (i.e. *Obp1* - KJ605390, *Obp2* - KJ605391, *Obp5* - KJ605392, *Obp6* - KJ605393, and *Obp7* - KJ605394) instead of the old names originally provided for all *Obp* predicted transcripts and proteins of the laboratory mouse.

### Protein structures

The protein PDBs were downloaded from RCSB Protein Data Bank (http://www.rcsb.org/) and visualized in a molecular visualization system PyMOL software v.1.5. (http://pymol.org). Examples of immunity-related proteins in the Results section are visualized on human homologs i.e. Cathelin-like domain of human Cathelicidin LL-37 (4EYC), Cystatin D (1ROA) and KLK1 (1SPJ) because no mouse structures exist to date. The last structure is an example of murine ESP1 (2LMK).

### Data availability

Proteomic data from LC-MS/MS are provided publically available in Supplementary Data file 1. RNAseq data from GS Junior are provided as Supplementary Data file 2.

## Results

### The saliva proteome

**Figure 1:**
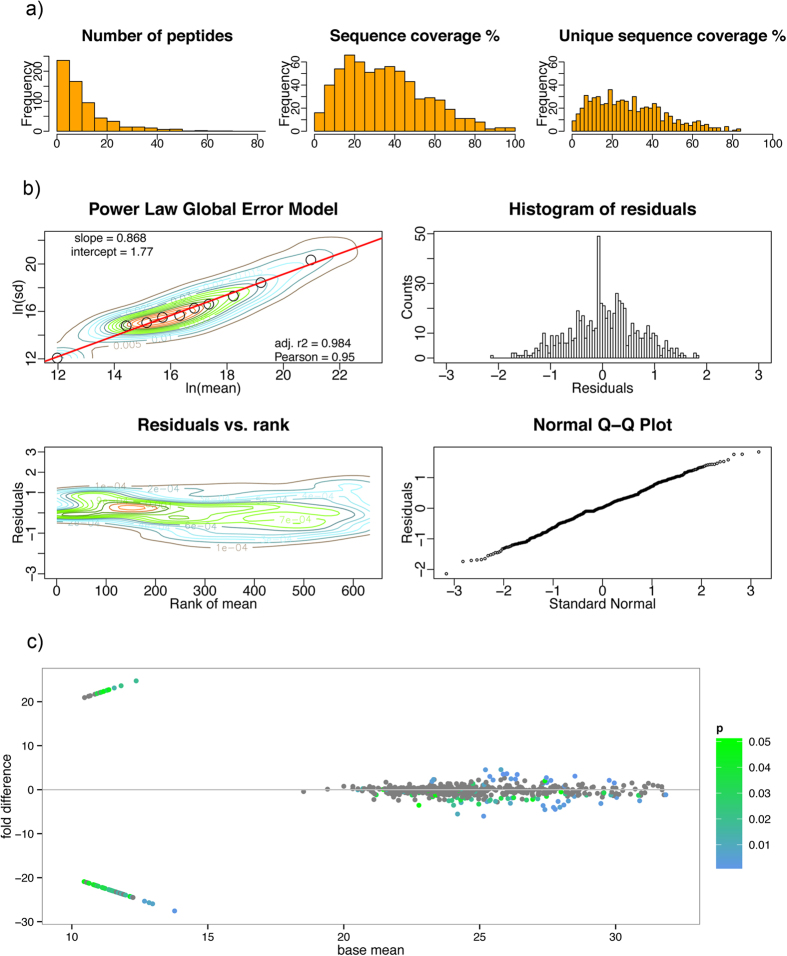
Details of the Power Law Global Error Model^47^: (**a**) histograms of the sequence coverage and the unique sequence coverage, (**b**) the model fitting on a female experimental condition, (**c**) MA plot with the differentially abundant proteins where significant points are colored from green (p < 0.05) to blue (p < 0.01).

We have generated the saliva proteome from the house mouse, *M. m. musculus* and detected a total of 633 proteins at 0.01 FDR (i.e. False Discovery Rate for all peptides and proteins). Successful identifications of these proteins resulted from a relatively high number of peptides per identification (11.33 ± 11.30, mean ± sd), sequence coverage (35.1 ± 20.2%), and unique sequence coverage (28.6 ± 17.8), Fig. 1a. Moreover, Spearman’s rank correlation between coverage and unique peptide coverage was high and significant rho = 0.74, S = 10866000, p-value < 2.2e-16 as well as the correlation between the number of MS/MS spectra and coverage (rho = 0.71, S = 12230000, p-value < 2.2e-16). Thus, the most abundant proteins have higher coverage and unique peptide coverage than those that were less abundant.

Having produced the saliva proteome we next performed the analysis of differentially abundant proteins between males and females using the Power Law Global Error Model (PLGEM)^47^. First of all, we calculated the signal-to-noise ratio - STN, because it explicitly takes unequal variances into account and because it penalizes proteins that have higher variance in each class more than those proteins that have a high variance in one class and a low variance in another^47^. Because PLGEM can only be fitted on a set of replicates of a same experimental condition we have done this for female data, Fig. 1b. Correlation between the mean values and standard deviations was high (r^2^ = 0.98, Pearson = 0.95) so we continued with the resampled STNs and calculated differences with corresponding p-values between males and females. We used the MA plot as a method of showing sex differences where fold differences are plotted against the base mean. Significant differentially abundant proteins are colored from green (p < 0.05) to blue (p < 0.01) in Fig. 1c.

PLGEM analysis of the level of sexual dimorphism revealed that 132 (21%) out of 633 identified proteins at 1% FDR and p < 0.05 were sexually dimorphic. Male biased proteins included 92 (14.5%) and female biased proteins included 40 (6.3%) successful identifications. Thus, the male-biased proteins were more common than the female-biased proteins in the saliva proteome of the house mouse subspecies *M. m. musculus*.

### The most abundant salivary proteins

Based on the median value we sorted our data to detect the most abundant proteins in the saliva proteome. We have filtered out potential contaminants such as keratins and also trypsins which are the enzymes that cleave all peptides before LC-MS in this study. The top five percent of the most abundant proteins included (i.e. in descending order): MUP6, BPIFB9B, ALBU, OBP5, OBP7, MUP5, CAH6, SCGB2B2, OBP1, ADA, SCGB1B2, AMY1, ACTB, PIP, LCN11, LACREIN, SCGB1B27, LCN13, KLK1KB9, OVOS, OBP2 (lipocalins are underlined). Out of these top abundant proteins, a third (i.e. seven proteins) was sex biased with male-biased OBP1, OBP2, LCN13, BPIFB9B, OVOS, AMY1 and female-biased KLK1B9.

### Lipocalins and other proteins involved in chemical communication

One of the most interesting results in our study is a finding that the saliva proteome is very rich of lipocalins belonging to different lipocalin sub-families and originating from several oro-facial expression sites. We have detected 20 (out of 55) mouse lipocalins belonging to the well annotated groups of LCNs (LCN2, LCN3, LCN4, LCN11, LCN12, LCN13, LCN14), OBPs (OBP1, OBP2, OBP5, OBP6, OBP7)^31^,^33^, and MUPs (MUP4, MUP5, MUP6, MUP8, MUP14, MUP17, MUP20, MUP21)^26^. A total of 10 lipocalins (50%) was significantly (p < 0.05) sexually dimorphic (OBP1, OBP2, LCN3, LCN4, LCN13, LCN14, MUP4, MUP8, MUP14, and MUP20). Only MUP8 was female biased (p = 0.026), while all other sexually dimorphic lipocalins were male biased. Furthermore, we have detected MUPs from the both earlier described phylogenetic groups (i.e. the ancestral group-A MUPs, and the later evolved group-B MUPs, refs 25,26) in the saliva. Moreover, OBPs and MUPs each belong to monophyletic groups of genes (bootstrap > 75) whilst LCNs are more heterogeneous and only LCN3, LCN4, LCN13, LCN14 form a monophyletic group previously detected in the mouse nasal and vomeronasal tissues. The complete mouse lipocalin phylogeny is provided elsewhere^27^,^33^.

The abundance of the male-biased MUP20 (p = 0.017) in the saliva was unexpected (Diagnostic peptides: FAQLSEEHGIVR, ENIIDLTNANR) because MUP20 has previously been detected only in the urine of male *Mus musculus domesticus* and C57BL/6. Therefore, we performed RNAseq-based analysis of the submandibular gland transcriptome to support this identification. We have detected the mRNA expression of several *Mup* genes including *Mup20, Mup4, Mup5,* and *Mup9*. However, all *Obp* members were absent in SMG transcriptome which supports our previous observations that OBPs are mainly expressed by the nasal (OE, VNO, NALT) and lacrimal glands/tissues^31^, and/or other as yet understudied oro-facial mouse glands. Similarly, male biased lipocalins LCN3 (VNSP1), LCN4 (VNSP2), LCN13 (OBP2A), LCN14 (OBP2B) are encoded by *Lcn* genes expressed by the nasal and vomeronasal tissues, but we detected them being highly abundant in the saliva proteome but not in the SMG transcriptome.

Along with lipocalins, we have also detected two male-biased exocrine glans-secreted peptides ESP1 (p = 0.006) and marginally ESP6 (p = 0.08), putative protein pheromones that are abundant in tears along with as yet uncharacterized lacrimal protein–Lacrein–which is also present in the saliva proteome of males and females but not in the transcriptome (SMG) in this study. Furthermore, we have also detected male-biased vomeromodulin (VOME, p = 0.003) in the saliva, however its expression site is also known to be exclusively the mouse vomeronasal organ^48^.

### Secretoglobins

In the saliva proteome of the mouse, we have detected 13 secretoglobin members with 7 of them being sexually dimorphic (i.e. male-biased: SCGB1B20, SCGB1B3, SCGB2A2, SCGB2B20, SCGB2B24, SCGB2B3, SCGB2B7) at p < 0.05. None of the salivary SCGB members was either male or female unique. On the level of SMG transcriptome, we have detected the expression of mRNA coding only three secretoglobins SCGB1B27, SCGB2B26, and SCGB2B27. The most abundant secretoglobin in the saliva proteome was a secretoglobin from family 2B, member 2 or SCGB2B2. However, we did not detect the mRNA coding SCGB2B2 in our trasncriptomic (SMG) data, thus, suggesting that SCGB2B2 (=ABPBG2) is also transported to the oral cavity from other tissues–most likely from the lacrimal or nasal glands/tissues^49^.

### Kallikreins and wound healing

**Figure 2:**
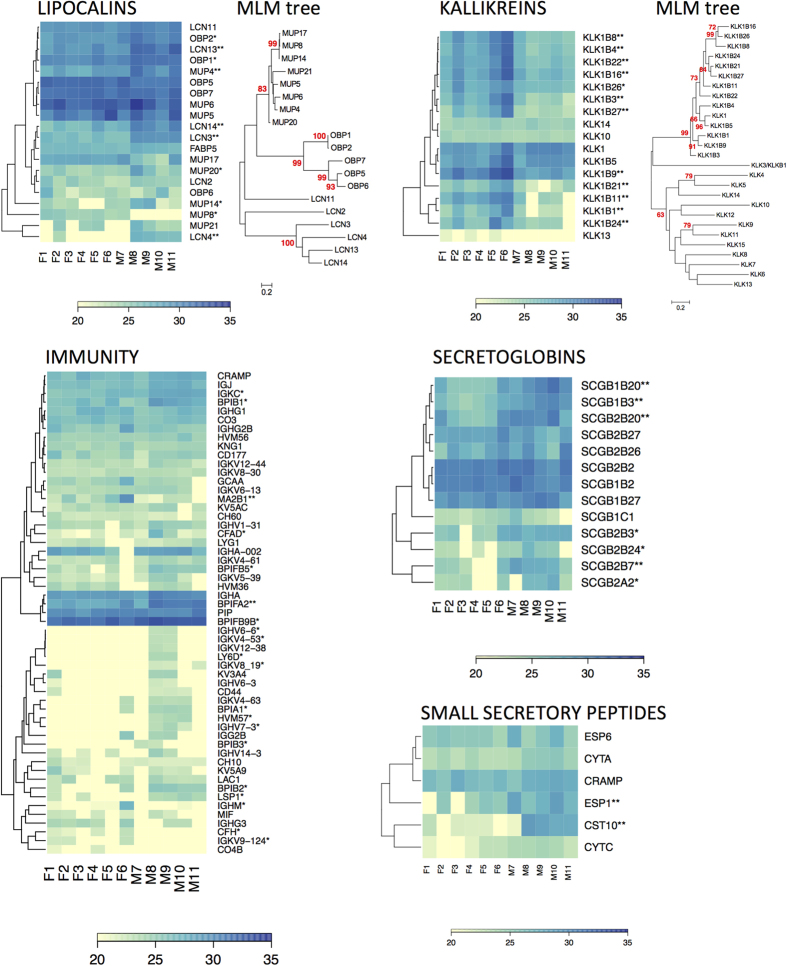
Graphical representation of the protein abundance values in heat maps shows sexually dimorphic proteins (labelled with stars: *P ≤ 0.05, **P ≤ 0.01, ***P ≤ 0.001) with notable variation between individuals. We provide the phylogeny dendrogram for kallikreins and a partial dendrogram for the detected lipocalins. The Maximum likelihood trees are showing the protein phylogeny based on the number of substitutions per site and with the bootstrap values. We consider a group of proteins monophyletic when bootstrap values are above 75.

Kallikreins are a group of serine proteases, which are capable of cleaving peptide bonds in various proteins also including some kallikreins. They have an antimicrobial activity and are involved in wound healing^50^. We have detected 4 kallikreins (KLK1, KLK10, KLK13, KLK14) and 13 kallikrein 1-related peptidases in saliva (KLK1B11, KLK1B16, KLK1B3, KLK1B1, KLK1B21, KLK1B22, KLK1B24, KLK1B26, KLK1B27, KLK1B4, KLK1B5, KLK1B8, KLK1B9). Kallikrein 1 and all kallikrein 1-related peptidases form a monophyletic cluster and it is notable in Fig. 2 that Kallikrein 1 is not an outgroup (i.e. the ancestral gene) to all other Kallikrein 1-related peptidases. Kallikreins KLK1, KLK10, and KLK14 were not sexually dimorphic whilst KLK13 was female biased but only marginally significant (p = 0.054) because it was detected only in three females. Almost all kallikrein 1-related peptidases were female biased (p < 0.01), except KLK1B5 (p = 0.08) that only revealed a trend. On the level of SMG transcriptome we have detected KLK1 and all Kallikrein 1-related peptidases. Furthermore, we have also detected angiotensinogen (ANGT) a substrate for KLK1 activity and CRAMP (i.e. Cathelicidin related anti-microbial peptide)–an antimicrobial peptide which is regulated by Kallikreins 5 and 7^50^.

### Proteins involved in innate immunity

Based on the functional classification and gene ontology, we have selected those genes that match our criteria, thus limiting the function to two keywords–immunity and antimicrobial. We have detected a total of 56 proteins fitting our criteria with 21 of them being significantly sexually dimorphic, Fig. 2. Additionally, we have identified 9 annexins equally expressed by individuals of both sex and which have strong effect upon the mechanism by which glucocorticoids (such as cortisol) inhibit inflammation.

Levels of sexual dimorphism are graphically represented in Fig. 2 with the full protein list provided in Data set 1. Interestingly, the immunity heat map in Fig. 2 shows rather low levels of the immunity-linked protein abundances except the three highly expressed ‘bactericidal permeability-increasing proteins’ (BPIB1, BPIFA2, and BPIFB9B) and one immunoglobulin (IGKC, Ig kappa chain C region). BPI proteins have an antibacterial activity against the gram-negative bacteria^51^. We have detected seven BPI members and all of them were male biased (p < 0.05): BPIA1, BPIB1, BPIB2, BPIB3, BPIFA2, BPIFB5, BPIFB9B. On the level of the mouse SMG transcriptome, however, we have detected only *Bpifa2* which has previously been detected as a transcript in the mouse parotid glands, and is also known as the parotid secretory protein - PSP^52^. Furthermore, BPIFA1 (PLUNC/SPLUNC1) and BPIFB1 (LPLUNC1) are known to be expressed by Bowman’s glands of the nasal passage^53^. Remaining members were most likely expressed by other oro-facial tissues (i.e. nasal, lacrimal, palatal, and salivary). Moreover, we have also detected CRAMP, a cathelin-related antimicrobial peptide, in the saliva of males and females.

## Discussion

**Figure 3:**
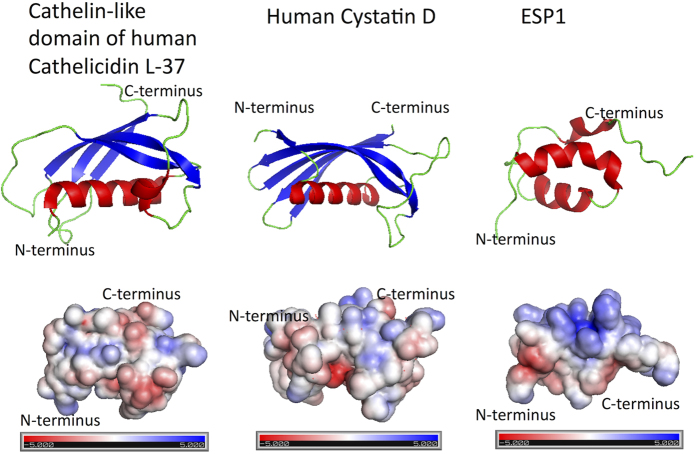
Graphical representation of structural and biochemical properties of murine salivary protein homologs - Cathelin-like domain of human Cathelicidin L-77, human Cystatin D and murine ESP1. Upper row: Cartoon representation of selected proteins where α-helices are in red, and beta sheets in blue color. Below are provided 3D representations of surface charge distribution of respective proteins, the charge scale is shown under each structure.

With the use of sensitive proteomic techniques, we show that saliva is a complex system containing chemical signal transporters, antibacterial and immunity linked proteins, and many other proteins that are involved in general physiology of the oral cavity. We also show that many nasal and lacrimal proteins are abundant in the saliva proteome, presumably as a consequence of their final transport to the oral cavity from tissues where they are expressed and where they function as VOC transporters. These include a group of odorant binding proteins (OBP) that we previously identified as predicted transcripts in the mouse genome^27^,^33^, detected their expression sites in various oro-facial tissues^31^, and finally detected them as proteins in the saliva proteome in this study. Because OBPs, MUPs and LCNs have similar tertiary structure (Fig. 3) with the capacity to transport VOCs, it is likely that together, nasal lipocalins, could be important for signal transduction but even more for a consequent neuronal desensitisation by transporting partially degraded VOCs to the oral cavity and then further to the digestive tract. However, it is in question why OBP1 and OBP2 are sexually dimorphic. It is possible that different levels of expression may reflect potential differences in the olfactory abilities between sexes. To further support the claim that these proteins originate only in the nasal and lacrimal tissues, it would help to analyse other independent oro-facial glands (i.e. parotid, sublingual, or von Ebner glands) and lymphoid tissues that are present in the oral cavity.

Because mice begin social interactions by investigating facial areas^54^ it is also likely that salivary proteins expressed by salivary glands along with those that were transported from the nose serve chemical communication together. It is however in question how would an individual benefit from having lipocalins with ligands that were inhaled from another individual. We suggest that a mixture of the self and of the other individual’s smell, that is spread on the receiver’s body during selfgrooming, could mediate peaceful social contacts between individuals within a deme–a structure typical for the house mouse social groups^55^.

Among MUPs, MUP20 or ‘Darcin’ was previously described as a protein pheromone that stimulates female attraction for particular *M. m. domesticus* males, improves spatial learning^56^,^57^, has been shown to function as an indicator of current health status of males^58^, and to predict the outcome of male-male territorial competition^3^. MUP20 expression levels are higher in dominant males during and prior to competition, making it predictive of dominance status^3^. However, we detected MUP20 being significantly male-biased (p = 0.017) but abundant in the saliva proteomes in individuals of both sex in *M. m. musculus*, and our RNAseq data revealed that MUP20 is coded by *Mup20* gene in the mouse submandibular gland. Thus, it is hard to imagine that this protein functions as a male-only pheromone (i.e. at least in this subspecies) if females produce such signal too. There is at least one study demonstrating that MUP20 is also present in the submandibular gland transcriptome of the laboratory mouse^25^. Therefore, previous studies describing MUP20 as a male-specific signalling protein that is present only in the urine of *M. m. domesticus*^56^,^57^ need to be further supported with more sensitive techniques. To add, we are also showing that MUP8 is among all MUPs the only one that is significant female biased. However, it remains to be determined where it is transcribed and translated.

Another protein that has been described as a male-specific signalling protein in the laboratory mouse is a 7 kDa protein, named as the exocrine gland-secreted peptide-1 or ESP1^59^,^60^. ESP1 is produced by the mouse lacrimal glands, secreted with tears and when experimentally transferred to the female vomeronasal organ, it stimulates V2R-expressing vomeronasal chemosensory neurons, and thus elicits an electrical response^60^. In *M. m. musculus* under this study, however, ESP1 and ESP6 were sex biased with the female levels being lower than those detected in males (Fig. 2). If ESPs cannot function as pheromones due to their occurrence in individuals of both sex, it is in question what are their other potential roles. One obvious role stems out of visualizing the electrostatics properties of ESP1 in Fig. 3. ESP1 has three α-helices with two helices being negatively charged and one (middle) being positively charged. This structural amphypathy fits the description of antimicrobial peptides (i.e. similar to CRAMP^61^). Thus it is possible that ESP1 and also other ESPs are involved in the host-defence against bacteria.

Secretoglobins (SCGBs or ABPs) were also suggested to play roles in chemical communication^62^. However, when experimentally tested, wild mice of the two subspecies did not show any difference in time spent sniffing urine to sniffing the urine with added ABPs^18^. In our data, some SCGBs/ABPs were male-biased but no member of this family was sex unique in the mouse saliva. Secretoglobins were previously detected in most body fluids and mucosa including lungs, uterus, nasal and oral cavities, and tears in many mammals including rabbits, mice, and humans. They are presumably involved in various processes including tissue repair, eye protection, and anti-inflammatory responses due to their capacity to transport various steroids^63^. Moreover, the PANTHER Overrepresentation test in this study did not identify any involvement in any known biological process of the mouse, which makes this family functionally understudied though an interesting system for future studies.

The saliva proteome also contains proteins that are involved in the regulation of harmonious symbiosis with bacteria and of potential risk of exogenous bacterial infection. A strategy called “nutritional immunity” prevents pathogens from acquiring the host iron^64^, which is an essential nutrient, but only small amounts of free iron are accessible. Therefore, bacteria acquire iron by a secretion of high-affinity iron sequestrating siderophores. The mammalian host, however, limits this process by the production of Lipocalin 2 (LCN2)^65^ which efficiently scavenges for catecholate-type siderophores (i.e. such as enterochelin, mycobactin)^66^. In our data the production of LCN2 (and also LCN11) was equal between sexes, thus suggesting that males and females similarly regulate such symbiosis with pathogens and/or the defence against them. Interestingly, when the lipocalin-acquired iron is transported to the oral cavity, which is the beginning of the digestive tract, the complex is in fact running towards the enzymatic digestion and thus iron is freed, and can presumably be used by symbiotic bacteria in the lower digestive tract. However, mammalian hosts evolved almost an array of other mechanisms of defence.

Other mechanisms of defence involve bactericidal proteins defending the mucosal layers of the body against pathogenic microbiota. In our data, we have detected seven members of the PLUNC (palate, lung, and nasal epithelium clone) protein family. These included bactericidal/permeability-increasing proteins^51^,^52^. All detected BPI proteins in the saliva proteome were male biased and at least three members were characteristic of being within the top ten of the most abundant salivary proteins (Fig. 2). It is possible that various antimicrobial proteins are male biased simply to compensate for the testosterone dependent immunosuppression of reproducing males^67^. Moreover the sex-dependent resistance against bacteria (*Salmonella typhimurium*) has also been demonstrated in the house mouse where males were more resistant than females^68^. We have also detected high levels of the prolactin-inducible protein (PIP) which is a submandibular gland protein with the ability to bind immunoglobulin G (IgG), IgG-Fc, CD4-T cell receptor, and different species of bacteria (mainly streptococci), thus playing an important role in non-immune defense^69^. A natural antibiotics CRAMP was detected in individuals of both sex. CRAMP forms an amphipathic alpha-helix similar to other antimicrobial peptides, whilst functional studies showed that CRAMP is a potent antibiotics against gram-negative bacteria by inhibiting the growth of a variety of bacterial strains^61^.

Kallikrein 1-related peptidases were female-biased in our data–a trend, described for the first time in wild mice. In the laboratory mouse, kallikreins seem to be male-biased and only the kallikrein 1-related peptidase b5 was female–biased^70^,^71^. These serine proteases are involved in the wound healing processes and have a strong antimicrobial activity^50^. Thus it is possible that the higher abundance of kallikrein 1-related peptidases in female saliva is adaptive as it may, for example, help females to maintain healthy skin development of their juveniles via kallikrein administration during a frequent allogrooming care. Moreover, we have also detected four chitinases (i.e. CH3L1, CHIA, CHIL3, CHIL4) in the saliva of males and females, which may aid removing the chitinous mouthparts of ectoprazites via administration of chitinases during the selfgrooming and allogrooming behaviour.

From the above list of proteins it is evident that saliva is a complex biological system that compromises between various functions including chemical communication, immunity and tissues repair. Because many lipocalins that we detected in the mouse saliva are known to be expressed by other tissues (e.g. nasal, lacrimal) it is likely that these proteins also act as scavengers that bind and excrete toxic compounds. We have already suggested that evolution of chemical communication and of the system of detoxification might have been driven by similar selective forces because both systems use the same pool of lipocalin transporters^27^,^31^. This hypothesis or as we call it the ‘toxic waste hypothesis’^27^ has later been suggested by another laboratory^72^ with the first experimental evidence provided in a recent paper^42^. They demonstrated that mice loaded with an industrial chemical, 2,4-di-tert-butylphenol (DTBP) use MUPs for a consequent detoxification^42^. Here we suggest that the nasal and olfactory lipocalins (including MUPs) transport potential toxic waste and various degradation products from chemical signals to the oral cavity, and further to the digestive tract where they are decomposed.

To conclude, we have provided the saliva proteome from wild-living individuals of the house mouse *Mus musculus musculus*. We aimed to identify the level of sexual dimorphism in the abundance of proteins that are involved in chemical communication because most studies focused on the western house mouse subspecies (*M. m. domesticus*) and on various inbred lines. Novelty of our findings includes the detection of sexually dimorphic proteins that were previously detected only in males with MUP20 and ESP1 being a good example. For the first time, we have also shown that the saliva proteome includes proteins that are produced mainly (but not exclusively) by olfactory tissues and which are presumably transported to the oral cavity. Altogether, that makes this system (saliva) interesting and an important source of chemical signals necessary for communication as well as an interesting source of multiple markers of physiological states.

## Additional Information

**How to cite this article**: Stopka, P. *et al*. On the saliva proteome of the Eastern European house mouse (*Mus musculus musculus*) focusing on sexual signalling and immunity. *Sci. Rep.* **6**, 32481; doi: 10.1038/srep32481 (2016).

## Supplementary Material

Supplementary Information

Supplementary Information
