# Supplementary material for: On the saliva proteome of the Eastern European house mouse (Mus musculus musculus) focusing on sexual signalling and immunity
Source: Sci Rep. 2016 Aug 31;6:32481. doi: 10.1038/srep32481 (PMC5006050; doi:10.1038/srep32481)

On the saliva proteome of the Eastern European house mouse (*Mus musculus musculus*) focusing on sexual signalling and immunity

Pavel Stopka*, Barbora Kuntová, Petr Klempt, Leona Havrdová, Martina Černá, & Romana Stopková

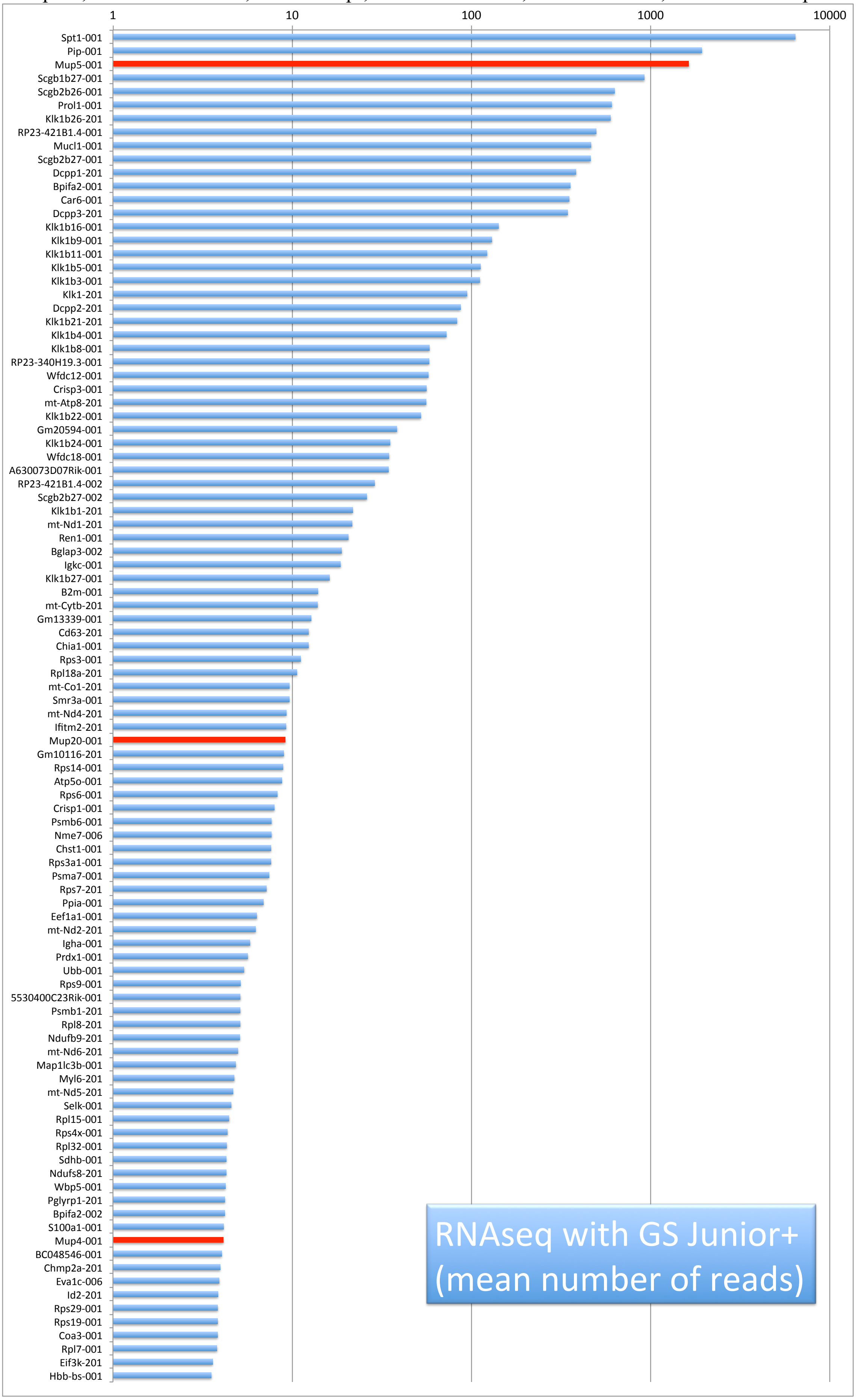

Supplement: Supplementary Information [file srep32481-s1.pdf]
